# Measuring extraordinary experiences and beliefs: A validation and reliability study

**DOI:** 10.12688/f1000research.20409.3

**Published:** 2020-05-14

**Authors:** Helané Wahbeh, Garret Yount, Cassandra Vieten, Dean Radin, Arnaud Delorme

**Affiliations:** 1 Research, Institute of Noetic Sciences, Petaluma, CA, 94928, USA; 2 Department of Neurology, Oregon Health & Science University, Portland, OR, 97239, USA; 3 Swartz Center for Computational Neuroscience, Institute for Neural Computation, University of California San Diego, La Jolla, CA, 92093-0961, USA

**Keywords:** paranormal, noetic, paranormal belief, paranormal experience, exceptional human experiences, anomalous information reception

## Abstract

**Background:** Belief in the paranormal is widespread worldwide. Recent surveys suggest that subjective experiences of the paranormal are common. A concise instrument that adequately evaluates beliefs as distinct from experiences does not currently exist. To address this gap, we created the Noetic Experiences and Beliefs Scale (NEBS) which evaluates belief and experience as separate constructs.

**Methods:** The NEBS is a 20-item survey with 10 belief and 10 experience items rated on a visual analog scale from 0-100. In an observational study, the survey was administered to 361 general population adults in the United States and a subsample of 96 one month later. Validity, reliability and internal consistency were evaluated. A confirmatory factor analysis was conducted to confirm the latent variables of belief and experience. The survey was then administered to a sample of 646 IONS Discovery Lab participants to evaluate divergent validity and confirm belief and experience as latent variables of the model in a different population.

**Results:** The NEBS demonstrated convergent validity, reliability and internal consistency (Cronbach’s alpha Belief 0.90; Experience 0.93) and test-retest reliability (Belief: *r = *0.83 *; *Experience: *r = *0.77). A confirmatory factor analysis model with belief and experience as latent variables demonstrated a good fit. The factor model was confirmed as having a good fit and divergent validity was established in the sample of 646 IONS Discovery Lab participants.

**Conclusions:** The NEBS is a short, valid, and reliable instrument for evaluating paranormal belief and experience.

## Introduction

“Paranormal beliefs pertain to phenomena that have not been empirically attested to the satisfaction of the scientific establishment” ^1^. Paranormal beliefs encompass a broad range of concepts, such as ghosts or spirits, extrasensory perception (ESP), extraterrestrial beings, and mind-to-mind communication, or telepathy. Belief in the paranormal is widespread around the world ^1– 13^. For example, in a Gallup poll of 1,002 United States adults conducted in 2005, 55% respondents believed in psychic or spiritual healing or the power of the human mind to heal the body, 41% believed in extrasensory perception, and 31% believed in telepathy or mind-to-mind communication ^14^.

However, having a belief in the paranormal does not necessarily mean having experienced the paranormal. A paranormal experience refers to an individual’s memory of an experience that one judges to be genuine. The memory of a paranormal experience relies on a different mental substrate than a belief based on environment, education and reasoning and the neural structures underlying memory of an experience and belief are likely different ^15, 16^. Paranormal belief and experience are often correlated when measured simultaneously, although this is rarely done ^17, 18^. For example, one study found a positive correlation ( *r* = 0.61) between paranormal experience and belief scores ^12^. Another interesting study found that exposure to television programs that regularly depict paranormal phenomena was positively correlated with belief, but only for respondents who had personal experiences ^19^.

Prevalence of reported paranormal experiences evaluated over the last 40 years in a variety of populations has ranged from a low of 10% in Scottish citizens ^20^ to a high as 97% in enthusiasts in the United States ^12^. Two very large prevalence studies have been conducted. One surveyed adults in 13 European countries and the United States (N=18,607). European respondents reported experiencing telepathy (34%), clairvoyance (21%), and contact with the dead (25%). Percentages for the U.S. adults were considerably higher: 54%, 25%, and 30% respectively ^21^. Another large study of British adults (n=4,096) found that 37% of respondents reported at least one paranormal experience defined as precognitions, extra-sensory perception, mystical experiences, telepathy, and after-death communication ^22^. Other smaller prevalence studies have been conducted around the world. Haraldsson *et al*. conducted two surveys of prevalence in Iceland, one in 1974 with 902 participants ^5^ and one in 2006 with 991 participants ^3^. He found that psychic phenomena increased from 59% of men and 71% of women in 1974 to 70% of men and 81% of women in 2006. In Scotland, 10–16% of the general population sample (n – 241) had experienced second sight, with the exception of the Grampian area where prevalence was more than doubled at 33% ^20^. Chinese, Japanese, African-American and Caucasian-American college students (n - 1922) were surveyed and 31–47% reported having at least one experience ^23^. Of 502 adults in Winnipeg, Canada 65% ^24^ and 38% of 622 Charlottesville, Virginia students and townspeople ^25^ reported having at least one experience. In the United States, 67% of the 1460 participants reported having had an ESP experience, 31% a clairvoyant experience, and 42% contact with the dead ^26^. More recently in the United States, 89.3% of the general population, 89.5% of scientists and engineers, and 97.8% of paranormal enthusiasts reported at least one paranormal experience ^12^.

Specificity of the work in this field is limited by the lack of questionnaires that adequately separate paranormal belief from experience and do so concisely ^1, 27^. Using ambiguous measures can lead to confounding the two constructs of belief and experience, and blur results ^1, 6^. Instruments that do separate these constructs are long and not conducive to the time constraints of many studies - see Exceptional Experiences Questionnaire ^28^ and Anomalous Experience Inventory ^29^. To address these limitations and as part of a larger research program on extended human capacities, we created the Noetic Experience and Belief Scale (NEBS), a 20-item survey that evaluates paranormal beliefs and experiences separately. The present studies investigate the psychometric properties of the Noetic Experience and Belief Scale in two populations. By studying these phenomena, we aim to gain a deeper understanding of the nature of consciousness and the reach of human potential.

The objectives of the following two observational studies were to evaluate the validity and reliability of the Noetic Experience and Belief Scale (NEBS) and to confirm the two latent variables of belief and experience in a confirmatory factor analysis. In study 1, the survey was administered to 350 participants for the validity and confirmatory factor analyses and again to a subsample of 96 of these participants for a test-retest analysis. In study 2, the survey was administered to a different population where divergent validity was evaluated and the factor model reevaluated. We hypothesized that NEBS would be valid, reliable, and demonstrate good fit for a model with belief and experience as latent variables in both populations.

## Methods

### Initial development of the NEBS

The NEBS was developed through consensus by the authors and two expert consultants who actively work in the field. This group was informed by our own previous studies and by reviewing other studies and previously used instruments that evaluated paranormal beliefs and/or experiences. One previous study ^30^ evaluated the prevalence of 27 paranormal experiences listed here in decreasing order of prevalence: Claircognizance, Clairempathy, Precognition, Lucid Dreaming, Emotional Healing, Clairvoyance, Clairsentience, Animal Communication, Telepathy, Aura Reading, Astral Projection, Clairaudience, Clairalience, Mediumship, Channeling, Physical Healing, Geomancy, Retrocognition, Psychometry, Remote Viewing, Automatic Writing, Clairgustance, Psychokinesis, Pyrokinesis, Levitation, and Psychic Surgery (please see extended data for definitions of each of these terms ^31^). Based on feedback from participants and a review of these items, we removed emotional healing (very similar to physical healing), psychic surgery (very rare), and clairsentience (very similar to claircognizance), renamed channel to psychophony and mediumship to contact with the dead, and added the item Information from Dreams. We then conducted another prevalence study in a different population. Notably, we did not use the jargon term for each paranormal belief/experience, but instead used as neutral language as possible to describe the experience itself. For example, rather than asking if the participant had ever experienced “pyrokinesis - the ability to create and/or manipulate fire”, the item asked “Have you ever created fire using only your concentration or will?” These neutral language items were then administered to participants consisting of three groups: a general population sample, scientists and engineers, and paranormal enthusiasts ^12^.

In both studies, we found that some items were highly correlated and represented overlapping constructs. They could also be viewed as specific nuanced experiences within a larger extended human capacities category. For example, psychic physical healing or the purported ability to feel other people’s physical symptoms in your own body and heal, transform, or transmute them would fall under the umbrella category of psychokinesis or the purported ability to influence a physical system without any physical interaction or with mental effort alone. Thus, in an effort to reduce participant burden and allow for quick assessment of experiences and beliefs we collapsed any overlapping constructs into individual items for each of the following categories: 1. Non-local consciousness (e.g. Astral Projection, Lucid Dreaming); 2. Extraterrestrials; 3. Precognition/Retrocausation; 4. Survival of Consciousness (after bodily death); 5. Contact with the dead (Mediumship); 6. Clairvoyance (Claircognizance, Clairempathy, Clairvoyance, Clairsentience, Aura Reading, Clairalience, Clairaudience, Geomancy, Clairgustance, Remote Viewing, Psychometry, Animal Communication); 7. Psychokinesis (Physical Healing, Psychokinesis, Psychic Surgery, Pyrokinesis, Levitation); 8. Telepathy; 9. Automatism (Channeling, Automatic Writing) 

We also reviewed a number of existing questionnaires that measured paranormal experience and/or belief ^8, 26, 28, 29, 32– 45^. A summary of this review is presented as Supplemental data A (please see extended data ^31^). Each questionnaire was evaluated for the number of items, whether it assesses belief, experience or both, whether it evaluates belief and experience as separate constructs, and subscales if applicable. From this review, an additional item on intuition, representing perhaps the most common paranormal experience, was added to the new scale for a total of 10-items.

The instrument was called the Noetic Experience and Belief Scale using noetic from the Greek noēsis/noētikos, meaning inner wisdom, direct knowing, or subjective understanding; and unlike a vague impression, a noetic experience carries a deep sense of authority and certainty. We included “noetic” in the title rather than “paranormal” in part because of the stigma associated with the term paranormal, which could introduce bias that might be mitigated by using an alternate term. Similarly, the paranormal categories were not stated in the scale but only descriptions of the constructs included (please see extended data) ^31^.

## STUDY 1: General population sample

### Procedures

The first study administered the NEBS to a randomly selected general population group in the United States to establish validity, test-retest reliability, and confirm the two latent variables of belief and experience. We contracted with Lucid, LLC (New Orleans, Louisiana) to obtain completed surveys from an unbiased census-distributed sample of 350 participants representative of the general population in the United States. The sample was unbiased in that it was not associated with the Institute of Noetic Sciences or any other paranormal or noetic-related group. Lucid, LLC is a marketplace that connects hundreds of sample suppliers with individual primary research studies to facilitate online surveys. Lucid uses screening questions to qualify respondents for a particular study then through programmatic technology aligns the best suppliers for that individual audience. Once a respondent qualifies through the screener, the appropriate suppliers are notified through an API and an email is triggered from the supplier directly to the survey taker. Each of the suppliers on the marketplace has approximately 200 pre-profiled mapped qualifications. These include age, gender, household income, job role, hobbies, etc. Lucid uses these qualifications as well as the screening questions to ensure efficiency and high quality when matching survey takers with individual projects. All potential volunteers are screened, checked for validity, and emailed a link to the survey. Participants were English-speaking adults in the United States. Inclusion criteria were: Adults aged 18 to 89, who could read and understand English, and were willing to complete questionnaires. Exclusion criteria were: Children (<18 years old) or Elders >89 years old. Elders 90 years old and older were excluded because the survey was designed to be anonymous and recording ages greater than 90 is considered private health information ^46^. Targets for distribution were based on United States census values and were as follows: Gender - 50% males and females, Age - 18–24 - 13%, 25–44 - 41%, 45–64 - 30%, 65+ - 16%; Ethnicity - Hispanic - 11%, Black - 12%, White (non-Hispanic) - 59%, Other - 18%.

The study was approved by the Institute of Noetic Sciences Institutional Review Board # WAHH_2018_06. Participants were given a link to a Health Insurance Portability and Accountability Act compliant survey on SurveyMonkey. The first page of the survey was a consent form (please see extended data ^31^). Participants were asked to read the form and check a box acknowledging that they had been informed of the procedures, and risks and benefits of participating in the study. They then completed the survey, which took approximately 15–20 minutes. Data were collected from November 9, 2018 through December 14, 2018. All data were collected anonymously, with no identifiers or IP addresses. Participants were compensated $3 for completing the survey once and $7 ($3 + $4) if they also participated in the retest administration. 

In total, 444 began the survey; 26 did not agree to the consent form and 57 agreed to the consent form but did not complete the survey. The remaining 361 participants completed the survey (underlying data ^47^). Surveys were collected between November 9, 2018 and December 13, 2018. Participants were on average 44 years old ± 16.8 and had 14.5 ± 5.3 years of education. Of these, 52% were female and 56.8% were in-relationship. Participants were mostly Caucasian (67% Caucasian, 13% Black or African American, 8% Hispanic or Latino, 6% Asian or Pacific Islander, 5% American Indian or Alaskan Native, and 2% preferred not to answer). In terms of salary, 67% of participants had earned between 0 and $75,000 (30% Under $30K, 37% $30K to under $75K, 11% $75K to under $100K, 11% $100K or under $150K, 7% $150K to under $250K, 3% $250K or greater, 2% Decline to answer) with an average household size of 2.6 ± 1.4.

Of the original 361 participants who completed the survey, 96 completed the same survey again approximately one month after the first administration (mean 35.3 days ± 3.7) between December 14, 2018 and January 2, 2019. Participants who completed the retest had similar demographics as the original sample (age 44 years old ± 16.9, education 14.3 ± 2.1, 54% male, 64% Caucasian, 53% in relationship, 74% with income under $75,000, and average household size of 2.4 ± 1.4).

### Measures

In addition to demographic information (e.g. age, gender, marital status, socioeconomic status), the main instrument in the survey was the *Noetic Experience and Belief Scale (NEBS).* The scale contains ten statements about beliefs in intuition, non-local consciousness, extraterrestrials, precognition, survival of consciousness, contact with the dead, clairvoyance, psychokinesis, telepathy, and automatism that all begin with the stem “I believe…” and then a description of the concept. The participant rates each belief statement on a slider anchored by Disagree Strongly (0) and Agree Strongly (100). For each of the ten items, participants also answered “I have personally had this experience.” on a slider scale anchored by Never (0) and Always (100). Two experience items were worded differently to accommodate the nature of the concept. The life after death experience item was worded “I have personally had an experience that I interpreted as a proof that consciousness survives the physical body.” and the contact with the dead item was worded “I have personally had the experience of contact with the dead.” Six of the 10 items were from the Australian Sheep-Goat Scale, three of which were exactly the same (#’s 9, 10, 11), and three were modified (#’s 4, 5, 14) ^45^. The scale results in overall scores for paranormal belief and experience by averaging the ten items for each subscale. Item scores can also be used individually for scores on each specific category. Internal consistency of the NEBS scale was calculated with a Cronbach α coefficient, as described subsequent sections (Full scale is available in extended data ^31^).

Convergent construct validity was measured by administering pre-existing survey instruments that evaluate similar concepts: *Australian Sheep-Goat Scale* ^45, 48^, *Revised Paranormal Belief Scale* ^43^, and *Anomalous Experiences Inventory (AEI)* ^29^. The *Australian Sheep-Goat Scale* is an 18-item questionnaire on various beliefs and experiences. Respondents endorse True (2 points), Uncertain (1 point), or False (0 points) for each item. Values are then summed to form a score ranging from 0-36. The *Revised Paranormal Belief Scale* is a 26-item scale that measures the degree of belief in the paranormal in each of seven dimensions: Traditional Religious Belief, Psi, Witchcraft, Superstition, Spiritualism, Extraordinary Life Forms, and Precognition. Respondents endorse how strongly they believe in each item on a 7-item Likert scale. Subscales and a total score are obtained by calculating means of specific items. The *AEI* is a 70-item questionnaire that evaluates multiple subscales: anomalous/paranormal experience, anomalous/paranormal beliefs, anomalous/paranormal ability, fear of the anomalous/paranormal, and drug use. Respondents answer True (1) or False (0) for each item and values are summed for each scale. The scales selected have already been assessed as valid and reliable and used in numerous peer-reviewed publications. Correlation matrices of the scores were evaluated for expected patterns of associations between measures of the same construct.

### Statistical methods

***Test-Retest.*** Some participants repeated the survey approximately one month later so that test-retest reliability could be assessed with a Pearson correlation coefficient.

***Sample size.*** Some sources suggest at least 10 people per item for psychometric validation although a recent review suggested that sample size is rarely justified *a priori* ^49^. We aimed for a sample size of 350 for the 20-item scale. For confirmatory factor analysis, there is also no agreement on the number of participants needed although sources ^50^ recommend approximately 10 participants for each estimated parameter (10 × 20 parameters = 200). We had 361 participants resulting in a ratio of 18.05 participants to each parameter estimated.

***Confirmatory factor analysis.*** A confirmatory factor analysis was used (rather than an exploratory factor analysis) because a theoretical framework was already established for evaluating belief and experience as separate constructs, albeit highly correlated ^1, 12^. The latent variables for the model were Belief and Experience. Observed variables were the 20 NEBS items. Univariate variables were tested for normality with the Shapiro-Wilk Test and any outliers assessed with scatter and box plots. Normality of residuals were evaluated with kernal density estimates and standardized normal probability plots. Outliers were evaluated with residuals, leverages, influence and Cook’s distance. Multicollinearity was evaluated with the variance inflation factor (VIF), which is the quotient of the variance in a model with multiple terms by the variance of a model with one term alone and quantifies the severity of multicollinearity. An unstructured covariance matrix was used so as to not impose any constraints on the variance and covariance values. All 20 items were highly correlated and thus, covariances between unique factors for all items were included in the model and then removed if they did not reach significance. All statistical analyses were conducted with Stata 15.0 (StataCorp, LLC, College Station, TX).

## Results

### Construct validity

The means and standard deviations for the paranormal belief and experience questionnaires are shown in Table 1. All correlation pairs were positive and significant at *p* = 0.05 level or less (all but three being more than *p* < 0.00005).

**Table 1.** Means, standard deviations, and Pearson *r* correlation coefficients for each questionnaire and subscales. Notes: *p* ≤ 0.0001 except for one shaded item where *p =* 0.02. With a Bonferroni multiple comparison adjustment, a significant *p-*value would be <0.0044 (0.05/16*16/2-16). Bold font represents Pearson *r* values that are moderately correlated (0.50-0.69), bold and underlined fonts represent values that are highly correlated (0.70-0.89), bold and double underlined fonts represent values that are very highly correlated (0.90-1.0). NEBS = Noetic Experience and Belief Scale, AEI = Anomalous Experiences Inventory.

		Mean ± SD	1	2	3	4	5	6	7	8	9	10	11	12	13	14	15	16
**1**	**NEBS Belief**	59.7 ± 21.9	1															
**2**	**NEBS Experience**	44.3 ± 25.6	**0.77**	1														
**3**	**Australian Sheep Goat**	18.2 ± 9.7	**0.80**	**0.68**	1													
**4**	**Paranormal Belief** **Scale Total**	4.1 ± 1.4	**0.78**	**0.65**	**0.79**	1												
**5**	**-Traditional** **Religious Belief**	5.1 ± 1.6	0.44	0.29	0.43	**0.65**	1											
**6**	**- Psi**	3.8 ± 1.5	**0.72**	**0.58**	**0.74**	**0.81**	0.38	1										
**7**	**- Witchcraft**	4.2 ± 1.8	**0.65**	0.49	**0.65**	**0.86**	**0.59**	**0.64**	1									
**8**	**- Superstition**	2.9 ± 1.9	**0.50**	**0.56**	0.48	**0.72**	0.33	0.44	**0.53**	1								
**9**	**- Spirituality**	4.1 ± 1.8	**0.76**	**0.61**	**0.77**	**0.90**	0.47	**0.76**	**0.74**	**0.58**	1							
**10**	**- Extraordinary Life** **Forms**	3.8 ± 1.7	**0.62**	**0.58**	**0.61**	**0.80**	0.34	**0.61**	**0.68**	**0.65**	**0.69**	1						
**11**	**- Precognition**	3.9 ± 1.8	**0.71**	**0.59**	**0.74**	**0.92**	0.48	**0.76**	**0.76**	**0.67**	**0.83**	**0.73**	1					
**12**	**AEI -Paranormal** **Experience**	8.5 ± 8.2	**0.65**	**0.69**	**0.72**	**0.67**	0.26	**0.54**	**0.57**	**0.60**	**0.62**	**0.62**	**0.66**	1				
**13**	**AEI -Paranormal Belief**	5.7 ± 3.5	**0.71**	**0.59**	**0.74**	**0.70**	0.35	**0.64**	**0.58**	0.42	**0.72**	**0.60**	**0.64**	**0.77**	1			
**14**	**AEI -Paranormal Ability**	3.5 ± 4.7	**0.58**	**0.67**	**0.59**	**0.60**	0.21	0.47	0.49	**0.64**	**0.53**	**0.59**	**0.57**	**0.91**	**0.65**	1		
**15**	**AEI -Paranormal Fear**	2.2 ± 2.0	0.34	0.33	0.35	0.38	0.22	0.20	0.31	0.41	0.33	0.35	0.35	**0.52**	0.36	**0.55**	1	
**16**	**AEI -Drug Use**	2.3 ± 2.0	0.42	0.46	0.42	0.43	0.12	0.32	0.38	0.43	0.37	0.43	0.43	**0.68**	**0.52**	**0.67**	0.36	1

### Reliability

***Internal consistency.*** Cronbach’s alpha was calculated for the NEBS Belief subscale items and Experience subscale items to measure the extent to which the items within the subscales correlated with each other and measured a similar construct ^51^. The ten belief items had a Cronbach’s alpha of 0.90 and average inter-item covariance of 429.9. The ten experience items had a Cronbach’s alpha of 0.93 and average inter-item covariance of 610.7.

*Belief:* On average, intuition, survival of consciousness, and non-local consciousness were the highest rated Beliefs (see means and standard deviations for each item in Table 2). All Belief construct pair correlations were significant ( *p* < 0.00005). Telepathy Belief and clairvoyance Belief were highly correlated ( Table 2; Very high: *r* = 0.90 to 1.00; High: *r* = 0.70 to 0.89; Moderate: *r* = 0.50 to 0.69; Low: *r* = 0.30 to 0.49; Negligible: *r* = 0 to 0.30 (Mukaka, 2012)). Many of the Belief pairs were moderately correlated.

**Table 2.** Means, standard deviations, and Pearson *r* correlation coefficients for each Noetic Experience and Belief Scale (NEBS) belief and experience item. Note: All values significant at *p* < 0.00005 except for belief in intuition and the experience of extraterrestrials ( *p* = 0.0004), contact with the dead ( *p* = 0.0001), psychokinesis ( *p* = 0.002), telepathy ( *p* = 0.002), and automatism ( *p* = 0.0016). Bold font represents Pearson *r* values that are moderately correlated, bold and underlined fonts represent values that are highly correlated.

	BELIEF	Mean ± SD	B1	B2	B3	B4	B5	B6	B7	B8	B9	B10	E1	E2	E3	E4	E5	E6	E7	E8	E9	E10
B1	**Intuition**	73.3 ± 20.3	1																			
B2	**Non-local** **consciousness**	67.6 ± 24.4	**0.54**	1																		
B3	**Extraterrestrials**	51.8 ± 32.5	0.26	0.37	1																	
B4	**Precognition**	58.6 ± 30.0	0.29	0.39	**0.58**	1																
B5	**Survival of** **Consciousness**	70.4 ± 32.0	0.25	0.30	0.25	0.36	1															
B6	**Contact with the** **Dead**	57.1 ± 33.4	0.32	0.34	**0.51**	**0.56**	0.46	1														
B7	**Clairvoyance**	58.6 ± 29.7	0.26	0.45	0.48	**0.69**	0.33	**0.60**	1													
B8	**Psychokinesis**	55.9 ± 30.1	0.24	0.44	0.47	**0.61**	0.39	**0.53**	**0.69**	1												
B9	**Telepathy**	54.4 ± 32.5	0.29	0.42	0.50	**0.65**	0.31	**0.62**	**0.70**	**0.64**	1											
B10	**Automatism**	49.3 ± 33.9	0.22	0.38	0.58	**0.67**	0.34	**0.67**	**0.68**	**0.64**	**0.68**	1										
	**EXPERIENCE**																					
E1	**Intuition**	69.5 ± 23.0	**0.56**	0.46	0.28	0.36	0.26	0.32	0.37	0.33	0.34	0.31	1									
E2	**Non-local** **consciousness**	60.9 ± 28.7	0.42	**0.64**	0.35	0.43	0.32	0.46	0.45	0.42	0.42	0.43	**0.59**	1								
E3	**Extraterrestrials**	29.1 ± 33.7	0.19	0.28	**0.53**	0.43	0.24	0.38	0.34	0.35	0.38	0.48	0.24	0.41	1							
E4	**Precognition**	42.4 ± 33.5	0.21	0.42	0.46	**0.68**	0.32	0.41	**0.52**	0.47	**0.50**	**0.57**	0.37	**0.53**	**0.65**	1						
E5	**Survival of** **Consciousness**	42.1 ± 35.6	0.22	0.36	0.45	0.41	0.44	0.46	0.40	0.39	0.40	**0.51**	0.23	0.46	**0.65**	**0.62**	1					
E6	**Contact with the** **Dead**	36.3 ± 35.4	0.21	0.37	**0.52**	0.46	0.27	**0.58**	0.46	0.49	**0.53**	**0.59**	0.33	0.43	**0.64**	**0.58**	**0.63**	1				
E7	**Clairvoyance**	42.4 ± 34.2	0.22	0.44	0.47	**0.54**	0.30	0.45	**0.63**	**0.52**	**0.55**	**0.55**	0.34	**0.50**	**0.60**	**0.70**	**0.62**	**0.63**	1			
E8	**Psychokinesis**	42.4 ± 33.4	0.19	0.42	0.44	**0.50**	0.38	0.43	**0.53**	**0.69**	**0.53**	**0.60**	0.33	0.48	**0.60**	**0.68**	**0.59**	**0.60**	**0.75**	1		
E9	**Telepathy**	43.5 ± 34.8	0.21	0.41	0.47	**0.55**	0.30	**0.51**	**0.57**	**0.52**	**0.79**	**0.61**	0.31	0.48	**0.56**	**0.64**	**0.57**	**0.65**	**0.70**	**0.65**	1	
E10	**Automatism**	34.5 ± 34.1	0.17	0.39	**0.50**	**0.51**	0.31	0.48	0.47	**0.52**	**0.53**	**0.69**	0.28	0.45	**0.72**	**0.70**	**0.65**	**0.69**	**0.74**	**0.76**	**0.68**	1

*Experience:* On average, intuition and non-local consciousness were the most common Experiences. All Experience pairs were significantly correlated ( *p* < 0.00005). Seven Experience pairs were highly correlated ( *r* = 0.70 - 0.89). Many Experiences were moderately correlated (r = 0.50-0.69).

*Belief and Experience:* Most Belief and Experience pairs were significantly correlated at the *p*<0.000005 level except for belief in intuition and the experience of extraterrestrials ( *p* = 0.0004), contact with the dead ( *p* = 0.0001), psychokinesis ( *p* = 0.002), telepathy ( *p* = 0.002), and automatism ( *p* = 0.0016). Belief in telepathy was highly correlated with the Experience of telepathy ( *r* = 0.79). Belief and Experience pairs of the same construct were all moderately correlated except for Survival of Consciousness which had a significant but low correlation. Many beliefs were moderately correlated with Experiences.

### Belief and Experience as separate constructs

**Figure 1.**
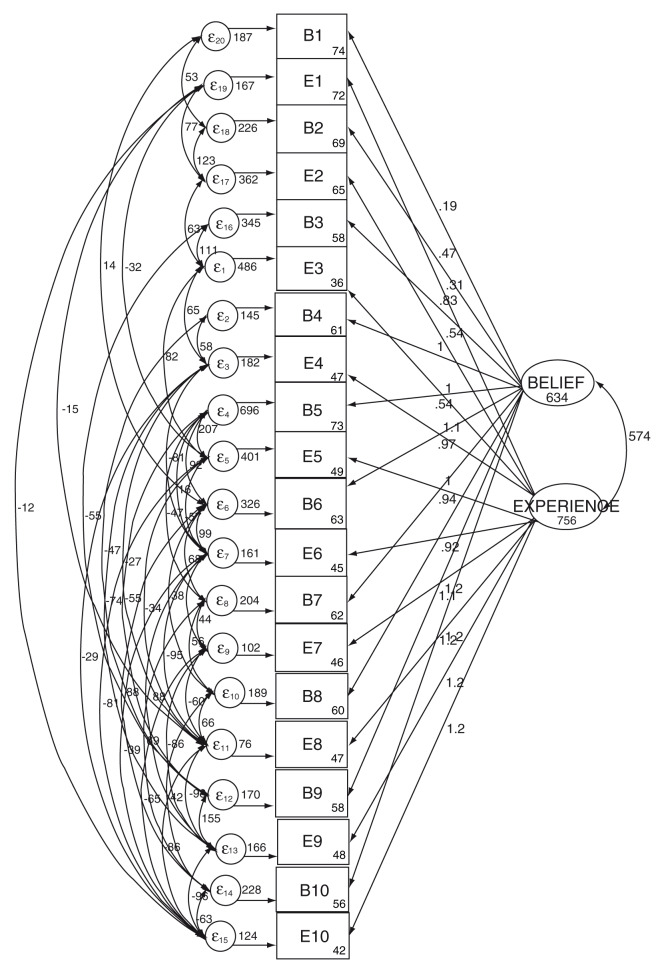
Theoretical and observed model of Noetic Experience and Belief Scale (NEBS) Belief and Experience. Significant covariances are denoted with curved arrows and error terms are displayed.

Confirmatory factor analysis was performed based on data from 361 respondents; there were no missing data. The retest data of the 96 participants were not included in the confirmatory factor analysis modeling. A correlation table of observed values with means and standard deviations is shown in Table 2. The *a priori* theoretical model of Belief and Experience items as described in the statistics section is presented in Figure 1.

We hypothesized a two-factor model to be confirmed in the measurement portion of the model where Belief and Experience were the latent variables. We evaluated the assumptions of univariate and multivariate normality and linearity. Univariate variables were not normally distributed individually. The ADF estimation method was used because it makes no assumption of joint normality or even symmetry for observed or latent variables (StataCorp, 2013). Residuals were normally distributed. There were no observations with a Cook’s distance greater than 1. No variable had a VIF less than 0.1 or greater than 10 (average VIF for all variables 3.29) indicating acceptable multicollinearity. The model chi-square (159) was 283.1 ( *p*<0.00005), the root mean square error of approximation (RMSEA) was 0.060 (90% confidence interval 0.051-0.069), the comparative fit index (CFI) was 0.94, the standardized root mean squared residual (SRMR) was 0.13, and the Tucker-Lewis fit index (TLI) was 0.90. These values represent a good fit of the model to the dataset as indicated by commonly reported fit statistics (RMSEA < 0.08, CFI ≥ 0.90, SRMR < 0.08, TLI ≥ 0.95) ^52^.

*Test-retest reliability:* The NEBS had high test-retest reliability for both the Belief ( *r =*0.83 *, p* <0.00005) and Experience ( *r =*0.77 *, p* <0.00005) subscales. The Wilcoxon sign-rank test was used to evaluate individual item and subscale differences because variables were not normally distributed. All items and subscale scores were not significantly different between the two time-points except for the telepathy Experience item which decreased Experiences for the second administration ( Table 3). Individual’s responses to the subscales remained relatively consistent across the repeated administration and above standardly accepted values for reliability of *r =*0.70 ^53^.

**Table 3.** Mean values and standard deviations for test-retest. B - Belief; E - Experience; NEBS - Noetic Experience and Belief Scale.

Item	Time 1	Time 2	*P-*value
**Intuition-B**	72.5 ± 18.3	73.9 ± 18.9	0.14
**Intuition-E**	70.0 ± 19.1	68.1 ± 21.5	0.79
**Non-local Consciousness-B**	67.9 ± 23.3	67.0 ± 24.1	0.64
**Non-local Consciousness-E**	62.2 ± 25.5	55.8 ± 29.9	0.15
**Extraterrestrials-B**	52.8 ± 31.0	53.5 ± 32.8	0.27
**Extraterrestrials-E**	27.5 ± 33.3	27.8 ± 34.1	0.96
**Precognition-B**	57.7 ± 28.0	56.9 ± 28.8	0.99
**Precognition-E**	40.1 ± 31.4	41.5 ± 33.9	0.30
**Survival of Consciousness-B**	70.3 ± 31.9	74.0 ± 30.7	0.12
**Survival of Consciousness-E**	42.5 ± 36.8	38.7 ± 35.2	0.40
**Contact with the Dead-B**	52.9 ± 32.8	51.5 ± 33.7	0.90
**Contact with the Dead-E**	33.1 ± 33.8	33.0 ± 34.5	0.77
**Clairvoyance-B**	57.9 ± 28.4	54.3 ± 30.8	0.90
**Clairvoyance-E**	42.5 ± 33.5	41.4 ± 32.5	0.95
**Psychokinesis-B**	55.1 ± 27.5	52.3 ± 30.6	0.55
**Psychokinesis-E**	41.7 ± 31.9	36.4 ± 32.9	0.24
**Telepathy-B**	54.1 ± 29.7	50.6 ± 31.1	0.87
**Telepathy-E**	41.4 ± 32.9	36.4 ± 31.9	0.05
**Automatism-B**	49.1 ± 33.1	45.3 ± 33.2	0.24
**Automatism-E**	34.2 ± 33.8	31.5 ± 32.8	0.16
**NEBS Belief**	59.0 ± 19.8	57.9 ± 21.5	0.37
**NEBS Experience**	43.5 ± 23.9	41.1 ± 26.2	0.16

## STUDY 2 – IONS Discovery Lab sample

### Methods

***Procedures.*** The NEBS was then administered to participants attending workshops at the IONS Discovery Lab and also online. Participants were recruited through workshop leaders hosting events at the IONS EarthRise Retreat Center in Petaluma, California and through workshop leaders hosting events at their own sites who contacted IONS to participate in the study. This study was approved by the IONS Institutional Review Board. Participants had to be adults (aged 18 years and above) with the ability to understand the consent form, were willing and able to complete the measures, and did not have an acute or chronic illness that precluded completion of the survey. Participants enrolled in the IONS Discovery Lab completed a number of surveys including the NEBS prior to their workshops. First, participants read and agreed to the consent form. Then data were collected anonymously through SurveyMonkey.

In total, 646 participants completed the surveys from March 17, 2018 through July 22, 2019 (underlying data ^47^). There were no missing data. Participants were on average 55.0 years old ± 13.3 with 17.4 ± 3.0 years of education, 75% female, 81% Caucasian, and 66% in relationship. In terms of income, 28% earned under $75,000, 16% between $75,000 and $100,000, 19% between $100,000 to $150,000, 15% between $150,000 to $250,000, and 12% above $250,000. The average household size was 2.3 ± 1.3.

***Measures.*** Relevant measures used to establish NEBS divergent validity, which tests whether concepts or measurements that are not supposed to be related are actually unrelated were: Arizona Integrative Outcomes Scale ^54^, Positive and Negative Affective Well-being Scale ^55^, single-item general health ^56^, acute sleep quality scale ^57^, the Numeric Pain Rating Scale ^58^, and Big Five Inventory-10 scale ^59^, and the compassion subscale of the Dispositional Positive Emotion Scale (1. It’s important to take care of people who are vulnerable; 2. When I see someone hurt or in need, I feel a powerful urge to take care of them; 3. Taking care of others gives me a warm feeling inside; 4. I often notice people who need help; 5. I am a very compassionate person.) ^60^.

Arizona Integrative Outcomes Scale (AIOS) is a one-item, visual analogue self-rating scale (VAS) with two alternate forms (one for daily ratings, AIOS-24h; and one for monthly ratings, AIOS-1m). The daily rating version was used for this study. The instructions are: "Please reflect on your sense of wellbeing, taking into account your physical, mental, emotional, social, and spiritual condition over the past 24 hours. Mark the line below with an X at the point that summarizes your overall sense of well-being for the past 24 hours." The horizontally-displayed VAS is 100 mm in length, with the low anchor being, "Worst you have ever been" and the high anchor being, "Best you have ever been." The AIOS has demonstrated the ability to discriminate between healthy and unhealthy populations and has adequate convergent and divergent validity ^54^.

Positive and negative affective well-being is measured with a variety of dichotomous indicators asking subjects whether they had experienced an emotional state for much of the day yesterday. For positive affect, the emotional states are happiness, enjoyment and smiling/laughter, which, aggregated together, have a reliability of α = 0.72. For negative affect, the emotional states are stress, worry and sadness, with a reliability of α = 0.65 ^55^.

Overall health is a single item question “In general, how would you rate your overall health?” which is answered by choosing one of five options: Poor; Fair; Good; Very good; Excellent ^56^.

Acute sleep scale is a single item scale asking participants to rate their quality of sleep over the past 24 hours on an 11-point numeric rating scale ranging from 0 denoting "best possible sleep" to 10 denoting "worst possible sleep" ^57^.

The Numeric Pain Rating Scale (NPRS) is a segmented numeric version of the visual analog scale in which a respondent selects a whole number (0–10 integers) that best reflects the intensity of his/her pain. The NPRS is anchored by terms describing pain severity extremes. Participants are asked to report pain intensity “in the last 24 hours” or an average pain intensity with 0 = “No pain” to 10 = “Worst possible pain” ^58^.

Big Five Inventory-10 (BFI) scale is a 10-item measure of the Big Five (or Five-Factor Model) dimensions: Neuroticism, Extraversion, Openness to Experience, Agreeableness, Conscientiousness. The BFI-10 was developed to provide a personality inventory for research settings with time constraints. It allows assessing the Big Five with only two items per dimension. Previous research has shown that the BFI-10 possesses psychometric properties that are comparable in size and structure to longer five factor inventories such as the NEO-PI-R which has 240 items. The score for each dimension is obtained by summing standard items and reverse scored items for each scale ^59^.

Compassion scale is 5 items from the Dispositional Positive Emotion Scale compassion subscale. It measures dispositional tendencies to feel positive emotions toward others in their daily lives. Items are rated from strongly disagree to strongly agree and scored from 1 to 7. Items are averaged for a total score and higher scores indicate greater levels of positive emotion ^60^.

***Statistical Analysis.*** Demographic information was qualitatively described for categorical variables. Means and standard deviations calculated for all continuous variables. Pearson correlations were conducted for relationships between measures. Cronbach’s Alpha was calculated for the Belief and Experience subscales. All analyses were conducted with Stata 15.0 (StataCorp, LLC, College Station, TX). The confirmatory factor analysis was conducted in the same was study 1.

## Results

The NEBS Belief items had a Cronbach’s alpha of 0.93 and an average inter-item covariance of 304.4. The NEBS Experience items had a Cronbach’s alpha of 0.91 and average inter-item covariance of 476.4. The experience scale was moderately correlated with the belief scale in this sample ( Table 4).

**Table 4.** Means, standard deviations and Pearson’s *r* correlation coefficients for NEBS and other questionnaires administered to IONS Discovery Lab sample. Notes: ^a^ = p < 0.00005; ^b^ = *p* < 0.001; ^c^ = *p* < 0.05; NEBS - Noetic Experience and Belief Scale. Bold font represents Pearson *r* values that are moderately correlated ( *r* = 0.50-0.69).

	Mean ± SD	NEBS Belief	NEBS Experience
**NEBS Belief**	81.0 ± 18.1		
**NEBS Experience**	59.4 ± 22.9	**0.64 ^a^**	
**Arizona Integrative** **Outcomes Scale**	65.8 ± 19.0	0.25 ^a^	0.23 ^a^
**Positive Affect**	0.9 ± 0.3	0.15 ^b^	0.26 ^a^
**Negative Affect**	0.5 ± 0.4	-0.10	-0.07
**Compassion**	5.7 ± 0.9	0.20 ^a^	0.21 ^a^
**Quality of Sleep (last** **24 hours)**	4.1 ± 2.5	-0.06	-0.09
**Pain (last 24 hours)**	3.0 ± 2.8	-0.11 ^c^	-0.07
**Extraversion**	3.3 ± 1.0	0.11 ^c^	0.14
**Agreeableness**	3.7 ± 0.8	0.16 ^a^	0.14 ^b^
**Conscientiousness**	4.1 ± 0.9	0.03	0.06
**Neuroticism**	2.6 ± 1.0	-0.21 ^a^	-0.21 ^a^
**Openness**	3.9 ± 0.9	0.23 ^a^	0.28 ^a^

Table 4 shows the correlations of NEBS Belief and Experience scale with the other measures administered. NEBS Belief had negligible correlations ( *r* = 0 - 0.30) with all items according to Mukaka *et al.* criteria except for NEBS Experience ^61^. NEBS Experience had negligible correlations (0 - 0.30) with all other scales (except NEBS Belief).

The same confirmatory factor analysis model developed through the general population data was applied to the IONS Discovery Lab dataset to evaluate its fit. The model chi-square (123) was 318.34 ( *p*<0.00005), the RMSEA was 0.060 (90% confidence interval 0.052-0.068), the CFI was 0.85, the SRMR was 0.24, and the TLI was 0.77. These values demonstrate a good fit of the model to this new dataset and similar to the original dataset where RMSEA = 0.060, CFI = 0.94, SRMR = 0.13, and TLI = 0.90.

## Discussion

The overall results of the two studies provide psychometric support for the validity and reliability of the NEBS as a brief assessment of self-reported paranormal beliefs and experiences. The participant demographics of study 1 reflected the general population of the United States as designated by the recruitment criteria. Construct validity of the NEBS Belief subscale was strong, as it was strongly correlated with multiple other scales measuring paranormal belief including the Australian Sheep Goat scale, the Psi, Spiritual and Precognition subscales of the Paranormal Belief Scale, and AEI Paranormal Belief subscale. Construct validity of the NEBS Experience subscale was also strong, demonstrating higher correlations to experience items such as AEI-Paranormal Experience and AEI-Paranormal Ability than other items such as Traditional Religious Beliefs. The NEBS did not measure paranormal fear or drug use as reflected in the low correlations on those AEI subscales. For divergent validity, there were only negligible correlations ( *r’s* between 0 and 0.30) to all other measures, providing more evidence that the NEBS is not measuring other constructs. Interestingly, other studies evaluating personality traits and paranormal beliefs have been mixed ^62, 63^, with some studies observing positive correlations with neuroticism ^64, 65^ (unlike our study which found a negligible negative correlation) and others not finding any correlations ^65– 67^. The NEBS reliability and internal consistency was also demonstrated through high Cronbach’s alphas for both subscales in two different samples. Our confirmatory factor analysis for two latent constructs of Belief and Experience in the general population dataset revealed a model good fit (RMSEA = 0.06), controlling for covariances between specific individual items, that was then confirmed with the IONS Discovery Lab sample (RMSEA = 0.06). RMSEA calculates the size of the standardized residual correlations and theoretically ranges from 0 (perfect fit) to 1 (poor fit). A model is considered satisfactory when RMSEA < 0.08 ^68, 69^. Our conceptual model of Belief and Experience as separate constructs and as evaluated through the NEBS was confirmed.

When measured separately, Belief and Experience are highly correlated. We found this in both of our samples (study 1: *r* =0.77; study 2: *r* = 0.64). Interestingly, the correlation was stronger in our general population sample than in our IONS Discovery Lab sample. The mean NEBS belief scores for the IONS Discovery Lab group were 21.3 points higher than the general population group (59.7 ± 21.9 general population vs. 81.0 ± 18.1 IONS Discovery Lab). The mean NEBS Experience scores were also greater in the IONS Discovery Lab group but only by 15.1 (44.3 ± 25.6 general population vs. 59.4 ± 22.9 IONS Discovery Lab). The AEI - Paranormal Belief and Paranormal Experience subscales were highly correlated in our study 1 sample as well (0.77). Interestingly, the original study of this scale found a much lower ( *r* =0.57) although significant correlation between the two subscales ^29^. We also found belief and experience to be highly correlated ( *r* = 0.61) for another mixed population of scientists and engineers, the general population, and paranormal enthusiasts ^12^. Other studies that have evaluated belief and experience in general have also found positive correlations ^17, 18^. A study examining the correlation between specific religious and classic paranormal beliefs, such as belief in heaven and hell or psychic healing, in relation to the paranormal experiences of illness cured by prayer and the use of the mind to heal the body, found mixed results. For example, belief in the devil and belief in illness cured by prayer had a low significant correlation ( *r =* 0.38), but the relationship between illness cured by prayer and the belief in psychic healing ( *r* = -0.04) was not ^40^.

Paranormal belief and experience are highly correlated in most studies that assess them, and yet they are distinctly different constructs that should be evaluated separately. What we do not yet understand is the causal or temporal nature of the relationship between belief and experience. Does paranormal belief precede experience or vice versa? Does someone’s belief in the paranormal prime them to experiencing it or does a subjective experience of the paranormal instill belief in the phenomena? Future longitudinal studies evaluating a baseline level of people’s beliefs and collecting data on how those beliefs change over time in relation to any experiences they have would be helpful in answering this question.

There are a number of limitations that should be kept in mind when reviewing the results of this study. The individual constructs included in the NEBS are highly correlated. Conceptually, the individual concepts are unique but could also be viewed as overlapping. For example, the items on non-local consciousness (B2. I believe that my consciousness is not limited by my physical brain or body. E2. I have personally had this experience.) and survival of consciousness (B5. I believe in life after death. E5. I have personally had an experience that I interpreted as a proof that consciousness survives the physical body.) could be considered as the same construct worded in a different way. The experience items are administered directly after the belief item of the same construct. The instrument was purposefully designed in this way to keep it concise. However, asking the belief question directly before the experience question could bias responses to the experience question in some way. We also acknowledge that the limited objective format of the survey (answered with a slider from 0-100) with constrained definitions is limiting. A more in-depth phenomenological approach would surely provide greater nuance and depth of understanding of belief and experience. However, the nature of such an instrument in terms of administration and scoring would not solve the problem of needing a simple and concise instrument. Others have suggested that paranormal beliefs stem from abnormal brain function or psychopathology such as Dissociative Identity Disorder or Schizophrenia. The NEBS focuses on the phenomenology of paranormal beliefs and experiences regardless of any pathology that may have generated them. Any NEBS results should be interpreted with these limitations in mind.

In summary, the NEBS is a 20-item survey rated on a sliding scale from 0-100, with 10 Belief and 10 Experience items. Both subscales demonstrated convergent validity, internal consistency, and test-retest reliability. A confirmatory factor analysis model demonstrated a good fit for Belief and Experience as separate latent variables. This model was confirmed in another sample where divergent validity was also established. The NEBS is a concise, valid, and reliable instrument for evaluating individual differences in paranormal beliefs and experiences. This measure provides a new tool for rigorously investigating these beliefs and experiences, and their relationship (as predictors, outcomes, or covariates) with other variables of interest such as psychological well-being, physical health, effects of interventions, coping with death and dying, grief and trauma resilience, and extended human capacities, just to name a few.

## Data availability

### Underlying data

Figshare: NEBS Validation Dataset. https://doi.org/10.6084/m9.figshare.9209510.v2 ^47^

This project contains the following underlying data:

- Wahbeh_NEBS Validation Dataset.xlsx (Excel workbook containing three datasets used in the NEBS validation study)

### Extended data

Figshare: NEBS Extended Data. https://doi.org/10.6084/m9.figshare.9770750.v2 ^31^

This project contains the following supportive material:

- Appendix 1 Noetic Experience and Belief Scale.docx (Noetic Experience and Belief Scale)
- Supplemental Data.docx (Table of previous studies and definitions of terms)
- Consent Form.pdf (consent form provided for participants)

Data are available under the terms of the Creative Commons Attribution 4.0 International license (CC-BY 4.0).
