# Supplementary material for: Measuring extraordinary experiences and beliefs: A validation and reliability study
Source: F1000Res. 2020 May 14;8:1741. Originally published 2019 Oct 11. [Version 3] doi: 10.12688/f1000research.20409.3 (PMC7256471; doi:10.12688/f1000research.20409.3)
Supplement: Peer review history

# Peer review: Reviewer response for version 3

**DOI:** 10.5256/f1000research.26440.r63414

**Published:** 2020-07-20

**Authors:** Christine Simmonds-Moore

**Affiliations:** 1 Department of Anthropology, Psychology and Sociology, College of Arts, Culture, and Scientific Inquiry, University of West Georgia, Carrollton, GA, USA

This paper describes two studies that sought to explore the reliability and validity of a new measure for measuring paranormal beliefs and experiences. The paper found that the measure displays reliability and validity. Following a CFA for both samples, there is support for the hypothesized existence of two sub-scales - one for belief and one for the experience. 

**Overall comments:**

 This measure is a valuable contribution to the existing literature that tends to examine beliefs and experiences separately or conflate experiences with beliefs in the measurement of both. However, there are some limitations. It is not currently clear which order the scales were presented to participants to ascertain the convergent construct validity in study 1. The inclusion of these other measures may have influenced responding to the NEBs if presented prior to the NEBS. This should be clarified herein. In terms of the measure itself,  think it would have been an improvement to randomly present items for the belief and experience versions of the same set of phenomena, and include more neutral language for the experience terms, which I think, still implicitly address beliefs and certainly conflate experience with appraisal, when this could have been further teased apart. Although this cannot be done on the current dataset, I think the authors should consider these issues in their discussion and note that the way that the items are worded and the measure was presented may have inflated the correlations between the 2 factors/subscales. I would also be interested to see the emergent results of EFA to see if the traditional distinctions between ESP, mind-matter interactions and survival play out within the dataset. The authors should also discuss the emergence of 2 factors for their CFA, as factors are statistical and not psychological. The belief questions and experience questions are framed as two parts to one overall type of experience, which immediately sets them apart as two things. I would like to see this addressed in their discussion with regard to how the measure will be developed moving forward. More detailed comments are included below. 

**Introduction:**

 I am not convinced that experience is necessarily a memory of an experience that one judges to be genuine. I think that there is evidence for folk to report an anomalous experience that could feasibly fit the category of paranormal without it necessarily being labeled/appraised as such and that this is demonstrated by research using the Irwin et al measure of anomalous experiences and appraisals. One might argue that all experience is being actively constructed as it is happening in addition to the memory of that experience. I think that the authors might think about adding another subscale (3rd part to each question) to future versions of this measure that reflects the experience without a paranormal attribution. This might be addressed in the discussion.

 I think that the relatively recent measure by Irwin *et al. *should be more thoroughly discussed in your literature review and more rationale given here for the development of the current measure as a viable alternative addition. The Irwin measure includes the possibility for several different appraisals of an ostensibly paranormal experience that is described phenomenologically rather than including a paranormal interpretation in the item description.

 It is commendable that the authors attempted to describe these phenomena without using the term paranormal, which may prime or bias responses to questions like this. The term noetic usually refers to experiential and direct knowledge, which I think encompasses experiences rather than beliefs which are usually understood to be more cognitive, but I think the terminology for the scale is fine as it is.

**Method:**

 A lot of good design and methodological considerations have been included herein. 

 This type of design is not usually referred to as an observational study in psychology. A more commonly used description would be a correlational design. 

 Who were the consultants who helped the authors develop the measure?  It seems that some of the items were derived from the existing literature which is commendable. With that said, it does seem as though you are including some items that are not usually included as traditional parapsychological experiences – i.e., extraterrestrials  - alongside others that are. This might be addressed later in terms of how the items behave as part of the questionnaire and which relate more to the overall measure/subscales.

 What was the order of presentation of the variables for study 1 and study 2? Ideally, NEBS should be first, so that responding was not influenced by scoring on the other paranormally relevant measures in study 1. 

 Although I think this is a valuable addition to the literature, I am not convinced that this measure completely addresses the very real problem of the separate measurement of beliefs and experiences of the same phenomena (although it is a step in the right direction). The items are presented together, with the belief item first. I think it would have been preferable to have presented the items in a random order overall and with the deployment of yet more neutral language and item construction. Many of the experience items are unfortunately conflated with belief because the paranormal belief always comes first (as the authors acknowledge in terms of possible response bias). In addition, the language of the experience item actually incorporates a paranormal interpretation (belief in something paranormal is followed by items asking if the respondent has experienced the paranormal phenomenon as denoted in the preceding belief item). This is problematic because the two subscales are more dependent from the outset than they might otherwise be. It would also be difficult for a skeptical person to respond to the experience items affirmatively because the experience cannot be interpreted neutrally or mundanely - in other words, the measure may be set up for believers to respond affirmatively to experiences. I think it would be preferable to have matched all the paranormal phenomena in terms of belief and equivalent experiences but used separate and neutral language for the experience items. Another way forward might be to add a 3rd part to the questions that would tap experiences (described neutrally, i.e., without any appraisal as part of the question).

 For example, although it is neatly presented with 2 parts to each paranormal phenomenon, it might work better to have the items presented in a more neutral way. E.g., some people have reported ____ which many interpret as ____ and then having a belief item and an experience item below it?  

 The authors should acknowledge this in their discussion and consider ways that they might adjust the measure in the future.

**Results:**

 Were the data for the first sample normally distributed? Do you have any data for gender breakdown, etc. to provide norms for these scales?

 Your Cronbach’s alpha is generally very high which is commendable - seems to be tapping something common and consistent - paranormal ideation.

 I think it would be valuable to explore individual items within each subscale as well as overall subscales for Cronbach’s alpha to explore how well each item performs. 

 As this paper seems to be focusing on the development of a new measure, I think it would be useful to spend some time on an item analysis and considering whether all of the items you have included in the measure should remain as part of the scale. An item analysis would usually look to see how the removal of a given item might influence the overall Cronbach’s alpha. Looking at the correlations between items, it does seem that some of your items perform less well than others – in particular the item describing intuition and possibly also the item referring to extraterrestrials. I suggest that this should be discussed in your results and discussion sections with some rationale given for why all of these items were retained. It may be that this was addressed in the prior studies conducted by and referred to by the authors, but it does seem that additional items were added prior to the current study (intuition). 

 I think it makes sense why a CFA has been run, but I would also like to see the results of an EFA  - perhaps as a footnote to explore emergent factors regarding how the different items work together (e.g., do we see belief and experience emerging here too, and do we see traditional categories of subjective paranormal phenomena emerging? Are they different for belief and experience?).

 The authors should further discuss the emergence of 2 factors for their CFA, as factors are statistical and not (necessarily) psychological. The belief questions and experience questions are framed as two parts to one overall type of experience, which immediately sets them apart as two things. I would like to see this addressed in their discussion with regard to how the measure will be developed moving forward. 

 Are there plans to explore how a longer period of time between the first and second mailings impact the test-retest data? One month is a fairly short period of time and I wonder how the measure would perform with a longer gap. 

 The second mailing included only 26% of those in the first mailing, which is on the low side, although it is noted that the demographics are equivalent. 

 It is commendable that the authors attempted to explore divergent reliability in the second sample. However, the second sample is very biased in terms of gender, age, affluence, etc. This should be noted in the discussion. The variables that you explored here do not relate to NEBS for this particular sample but may relate to other more representative samples. Which order were the scales presented in? Was NEBS first or after these other measures? The second sample seems to be very different to the first as you note that experience and beliefs are both much higher in this second sample.

**Discussion:**

 The questionnaire has potential but has some limitations. The authors should talk more about how the measure might be developed/tested in the future.  The authors include a very brief discussion on the relationship between experiences and beliefs in their discussion. There is a lot of research on this topic, particularly in terms of recent research on appraising ostensibly paranormal events as being at the heart of the generation of beliefs. I think that Pekala’s (2000) work on the levels of anomalous experiences would be a valuable addition here or in the literature review, as they talk about anomalous sensations, anomalous experiences, and anomalous beliefs and how they relate to one another. They note that it is very possible to have anomalous sensations and experiences in the absence of holding paranormal beliefs and vice versa and this should be included in the paper.

Is the work clearly and accurately presented and does it cite the current literature?

Yes

If applicable, is the statistical analysis and its interpretation appropriate?

Partly

Are all the source data underlying the results available to ensure full reproducibility?

Yes

Is the study design appropriate and is the work technically sound?

Yes

Are the conclusions drawn adequately supported by the results?

Yes

Are sufficient details of methods and analysis provided to allow replication by others?

Yes

Reviewer Expertise:

Paranormal beliefs and disbeliefs; exceptional experiences.

I confirm that I have read this submission and believe that I have an appropriate level of expertise to confirm that it is of an acceptable scientific standard.

# Peer review: Reviewer response for version 3

**DOI:** 10.5256/f1000research.26440.r63412

**Published:** 2020-05-28

**Authors:** Rudiger Seitz

**Affiliations:** 1 Heinrich-Heine-University Düsseldorf, LVR-Klinikum Düsseldorf, Düsseldorf, Germany; 2 Florey Neuroscience Institutes, Melbourne, Australia

The authors responded comprehensively to the comments and made corresponding changes in the manuscript.

 As the authors agree, however, that E-statements are essentially B-statements and that this is intrinsic in all phenomenological surveys that ask participants to reflect on their experiences, it remains unclear why it is stated in manuscript that the aim of the study was to to differentiate beliefs from experiences rather than to focus on the validity and reliability of the scale.

Is the work clearly and accurately presented and does it cite the current literature?

Partly

If applicable, is the statistical analysis and its interpretation appropriate?

Yes

Are all the source data underlying the results available to ensure full reproducibility?

Yes

Is the study design appropriate and is the work technically sound?

Yes

Are the conclusions drawn adequately supported by the results?

Partly

Are sufficient details of methods and analysis provided to allow replication by others?

Yes

Reviewer Expertise:

Neurology, Cognitive Neuroscience

I confirm that I have read this submission and believe that I have an appropriate level of expertise to confirm that it is of an acceptable scientific standard.

# Peer review: Reviewer response for version 2

**DOI:** 10.5256/f1000research.24741.r60396

**Published:** 2020-04-28

**Authors:** Malcolm Schofield

**Affiliations:** 1 Department of Psychology, University of Derby, Derby, UK

I want to thank the authors for the changes they made to the paper and their thoughtful comments regarding the lack of an EFA on the first sample. The suggestion of an EFA was simply a methodological consideration further strengthen the paper; I am aware that an EFA is not always considered to be the best way of confirming a model when theory strongly suggests a particular factor structure. However, it can introduce unexpected results that can contribute to the current theory. I also apologise for my unfortunate use of the term ‘this would probably not give the desired outcome’. I also take the point ‘If a CFA fits well and satisfies all assumptions but an EFA indicates that the underlying structure of the data can be represented by different factors, is this sufficient evidence to discard the original CFA’ is not sufficient evidence to discard the CFA. I am happy to accept the paper based on the current changes, but I still feel that the way the experience items are worded and presented is a weakness with this paper and that an EFA could provide some interesting results in terms of theory behind how the relationship belief and experience is conceptualised. However, this paper does provide a much-needed way of measuring both belief and experience.

Is the work clearly and accurately presented and does it cite the current literature?

Partly

If applicable, is the statistical analysis and its interpretation appropriate?

Partly

Are all the source data underlying the results available to ensure full reproducibility?

Yes

Is the study design appropriate and is the work technically sound?

Partly

Are the conclusions drawn adequately supported by the results?

Yes

Are sufficient details of methods and analysis provided to allow replication by others?

Yes

Reviewer Expertise:

Paranormal belief

I confirm that I have read this submission and believe that I have an appropriate level of expertise to confirm that it is of an acceptable scientific standard.

# Peer review: Reviewer response for version 2

**DOI:** 10.5256/f1000research.24741.r61785

**Published:** 2020-04-03

**Authors:** Rudiger Seitz

**Affiliations:** 1 Heinrich-Heine-University Düsseldorf, LVR-Klinikum Düsseldorf, Düsseldorf, Germany; 2 Florey Neuroscience Institutes, Melbourne, Australia

This is the careful revision of a thorough study on a 20-item questionnaire for assessing paranormal beliefs using a visual analogue scale with 10 items on belief and 10 items on experience. The test was applied to 361 subjects in an observational study and to a second group of 646 control subjects recruited in their lab. Statistical tests were used to determine validitiy, reliability, internal consistency as well as test-retest reliability. The results show that the questionnaire matches these quality measures. The study is novel and timely. But there still is room for improvement.

 The authors call their scale noetic. Noetic refers to the philosophical concepts of mind, understanding or intellect and was elaborated as a notion of high-level scientific thinking in the nineteenth century. The authors quote the ancient ethymology on page 4. Accordingly, the title of the study in its present form suggests a broad belief concept comprising both natural and potentially paranormal beliefs. Since the authors treat noetic, however, synonymous to paranormal and since paranormal beliefs are the content of the paper, noetic is misleading for the title of this paper and ought to be replaced by e.g. paranormal.

 Likewise, the authors fail to provide a greater picture of what a belief is. In fact, natural or normal beliefs are essential products of brain function (Seitz *et al. *in this journal; cited in the paper). In contrast the paranormal beliefs of the kind the authors address in this study are considered to result from abnormal brain function. As paranormal beliefs are the objective of the study, the questionnaire is not applicable as a general or comprehensive belief scale. This should be stated in the discussion.

 Right from the first sentence of the introduction the authors point out that they focus on “paranormal beliefs”. Also, the authors suggest that belief pertains to the abnormal or paranormal and provide a good survey about similar studies. But it remains unclear what abnormal beliefs are in comparison to delusion-like beliefs as investigated in the normal population by Pechey and Halligan (2011).

 The scale items (presented in Appendix I) consist of statements beginning with “ I believe …” (B-statement) and throughout with one statement “I have personally had this experience” (E-statement). In contrast to what the authors note the high correlation of the ten B- and E-statements is not surprising, as both statements involve the same types of neural information processing. The B-statements reflect a personal inference explaining a previous perception or experience of high personal relevance, which is a belief as described earlier by Seitz and Angel in this journal (cited in the paper). Therefore, it is justified that the authors call these phrases B-statement. For comparison, the E-statement focuses semantically onto an experience of a paranormal perception. Asking the subjects to approve or deny this statement, however, requires that the subjects recall this very perception or experience. This recall from memory has a probability of being true for the subject which also reflects a belief and would be expressed correspondingly by “I believe that I have personally had this experience”. Consequently, this renders the E-statements to be essentially B-statements as well.

 The authors present nicely the development of their scale (pages 3 through 4). This should go under a headline of its own. For comparison, study 1 refers to the procedures described on pages 4 through 5. Therefore, the headline of study 1 should be moved accordingly.

 It is unclear who the 899 participants are (page 4 left column). If this pertains to both study populations, why is this number mentioned here? Please, clarify. Likewise, it is unclear, in which subgroups these participants were analyzed.

 Page 12, left column, line 1: I guess it should be than instead of that.

Is the work clearly and accurately presented and does it cite the current literature?

Partly

If applicable, is the statistical analysis and its interpretation appropriate?

Yes

Are all the source data underlying the results available to ensure full reproducibility?

Yes

Is the study design appropriate and is the work technically sound?

Yes

Are the conclusions drawn adequately supported by the results?

Partly

Are sufficient details of methods and analysis provided to allow replication by others?

Yes

Reviewer Expertise:

Neurology, Cognitive Neuroscience

I confirm that I have read this submission and believe that I have an appropriate level of expertise to confirm that it is of an acceptable scientific standard, however I have significant reservations, as outlined above.

# Response

**Published:** 2020-05-07

**Authors:** Helané Wahbeh

**Affiliations:** Oregon Health & Science University, USA

We thank Dr. Seitz for his thoughtful review of our paper. We have included his comments below with our response in italics.

RS: The authors call their scale noetic. Noetic refers to the philosophical concepts of mind, understanding or intellect and was elaborated as a notion of high-level scientific thinking in the nineteenth century. The authors quote the ancient ethymology on page 4. Accordingly, the title of the study in its present form suggests a broad belief concept comprising both natural and potentially paranormal beliefs. Since the authors treat noetic, however, synonymous to paranormal and since paranormal beliefs are the content of the paper, noetic is misleading for the title of this paper and ought to be replaced by e.g. paranormal.

*- We have changed the title of the paper to “Measuring extraordinary experiences and beliefs: A validation and reliability study.”*

RS: Likewise, the authors fail to provide a greater picture of what a belief is. In fact, natural or normal beliefs are essential products of brain function (Seitz et al. in this journal; cited in the paper). In contrast the paranormal beliefs of the kind the authors address in this study are considered to result from abnormal brain function. As paranormal beliefs are the objective of the study, the questionnaire is not applicable as a general or comprehensive belief scale. This should be stated in the discussion.

*-We have added these sentences to the discussion “Others have suggested that paranormal beliefs stem from abnormal brain function or psychopathology such as Dissociative Identity Disorder or Schizophrenia. The NEBS focus on the phenomenology of paranormal beliefs and experiences regardless of any pathology that may have generated them.”*

RS: Right from the first sentence of the introduction the authors point out that they focus on “paranormal beliefs”. Also, the authors suggest that belief pertains to the abnormal or paranormal and provide a good survey about similar studies. But it remains unclear what abnormal beliefs are in comparison to delusion-like beliefs as investigated in the normal population by Pechey and Halligan (2011).

*-Pechey and Halligan comment in their abstract that “Delusions are defined as false beliefs different from those that almost everyone else believes.” Their study shows that many of these beliefs are commonly believed by the general population and thus, should not be considered delusions. In fact, their item “The soul or spirit survives death” corresponds with the NEBS item #2 and “Some people communicate with the dead” corresponds to NEBS item #6 and both these items were commonly endorsed (@64% and 55% respectively) in Pechey and Halligan’s study. We also never use the word “abnormal” in the paper. We are not saying that paranormal beliefs are abnormal anywhere in the manuscript. As Pechey and Halligan’s paper supports, some of these beliefs are “normal” in that many in the population believe them. Also, the first DSM-5 schizophrenia diagnosis criterion revolves around the symptoms that may include delusions but do not have to. There must be two or more symptoms of delusions, hallucinations, disorganized speech, grossly disorganized or catatonic behavior, or negative symptoms for at least a one-month (with at least one of those symptoms being delusions, hallucinations, or disorganized speech). Most importantly in this conversation is that the symptoms must impair the person’s work, interpersonal relations, or self-care for a significant amount of time since the symptoms began. The symptoms must also be ongoing for at least 6 months and other mental illness and effects from substances or medical conditions must be ruled out. Other symptoms that contribute to a schizophrenia diagnosis beyond these key criteria include: inappropriate emotions, disturbed sleep, negative mood, anxiety and phobias, detachment or feeling of disconnection from self, a feeling that the surroundings aren’t real, impairments in language, cognitive processing and memory, social deficits, and hostility and aggression.*

*The goal of our study was not to highlight pathology or abnormal function but to provide a tool to quickly evaluate the phenomenology of experience and beliefs of this nature.*

RS: The scale items (presented in Appendix I) consist of statements beginning with “ I believe …” (B-statement) and throughout with one statement “I have personally had this experience” (E-statement). In contrast to what the authors note the high correlation of the ten B- and E-statements is not surprising, as both statements involve the same types of neural information processing. The B-statements reflect a personal inference explaining a previous perception or experience of high personal relevance, which is a belief as described earlier by Seitz and Angel in this journal (cited in the paper). Therefore, it is justified that the authors call these phrases B-statement. For comparison, the E-statement focuses semantically onto an experience of a paranormal perception. Asking the subjects to approve or deny this statement, however, requires that the subjects recall this very perception or experience. This recall from memory has a probability of being true for the subject which also reflects a belief and would be expressed correspondingly by “I believe that I have personally had this experience”. Consequently, this renders the E-statements to be essentially B-statements as well.

*-Yes, this is a good point. We believe this is intrinsic in all phenomenological surveys that ask participants to reflect on their experiences.*

RS: The authors present nicely the development of their scale (pages 3 through 4). This should go under a headline of its own. For comparison, study 1 refers to the procedures described on pages 4 through 5. Therefore, the headline of study 1 should be moved accordingly.

*-Thank you we have moved the Study 1 heading to below the development of the scale.*

RS: It is unclear who the 899 participants are (page 4 left column). If this pertains to both study populations, why is this number mentioned here? Please, clarify. Likewise, it is unclear, in which subgroups these participants were analyzed.

*-Thank you, we see how this can be confusing. We were listing the number of participants in the referenced study. We’ve removed this from the manuscript as the number of participants in this referenced study is not important here. Readers can see the referenced paper if they would like to see more details of participant numbers in each group.*

Page 12, left column, line 1: I guess it should be than instead of that.

*-Thank you, this has been fixed.*

# Peer review: Reviewer response for version 1

**DOI:** 10.5256/f1000research.22429.r56127

**Published:** 2020-01-17

**Authors:** Malcolm Schofield

**Affiliations:** 1 Department of Psychology, University of Derby, Derby, UK

This paper acknowledges the importance of having a scale that distinguishes paranormal belief from paranormal experience and develops a concise measure of twenty items. However, to bring it up to an acceptable standard, the following points should be addressed:

- The introduction is severely lacking any review of previous scales and how different factors have been conceptualised in the past.
- The development of the NEBS should be in the methods section and not the introduction.
- The methods sections would benefit from traditional subheadings.
- The results section contains demographic data which should be in the participant's section in the methods.
- An exploratory factor analysis would have been useful on the first sample.
- For study one, the CFA should come first followed by test retests.
- Double-check the fit indices (particularly the CFI, the cut of is listed as .90 when in fact it should be .95) and was the chi-square significant?
- Study two – why are there wellbeing measures? They are only mentioned briefly via a table in the results.
- The items relating to experience are all the same.
- The participant is asked if they have experienced something directly after they have been asked if they believe it; this could present a confound.

Overall, while this paper offers an interesting premise, there are several flaws. The scale itself does not have dedicated experience items that refer to specific phenomena, which is problematic. The methodology itself is also flawed. I suspect an Exploratory Factor Analysis (EFA) would reveal that the factors would be around the phenomena rather than the dichotomy of belief and experience. I.e., a person, who believes in particular phenomena is more likely to experience them; therefore, the factors will demonstrate this. I would recommend that this be more of an exploratory study and that an EFA be run on the first sample at the very least. However, this would probably not give the desired outcome. The items that relate to experience should ideally be able to be answered in isolation and not be dependent on the belief items. This could act as a prime, with people who state they believe in something when asked if they have experienced it directly after being more likely to agree.

Is the work clearly and accurately presented and does it cite the current literature?

Partly

If applicable, is the statistical analysis and its interpretation appropriate?

Partly

Are all the source data underlying the results available to ensure full reproducibility?

Yes

Is the study design appropriate and is the work technically sound?

Partly

Are the conclusions drawn adequately supported by the results?

Yes

Are sufficient details of methods and analysis provided to allow replication by others?

Yes

Reviewer Expertise:

Paranormal belief

I confirm that I have read this submission and believe that I have an appropriate level of expertise to confirm that it is of an acceptable scientific standard, however I have significant reservations, as outlined above.

# Response

**Published:** 2020-02-12

**Authors:** Helané Wahbeh

**Affiliations:** Oregon Health & Science University, USA

We thank the reviewer for their thoughtful comments. We have made revisions to the paper as a result that we believe have strengthened it. Below you will find each reviewers comments and our changes to the manuscript or responses. 

The introduction is severely lacking any review of previous scales and how different factors have been conceptualised in the past.

*- The original manuscript included Supplementary Data that summarized a review of 18 previous scales that informed the design of our scale. We have revised the text in the Introduction to clarify and draw attention to this. The revised text reads: "We also reviewed a number of existing questionnaires that measured paranormal experience and/or belief (8, 26, 28, 29, 32– 45 ). A summary of this review is presented as Supplemental data A. (please see extended data (31)). Each questionnaire was evaluated for the number of items, whether it assesses belief, experience or both, whether it evaluates belief and experience as separate constructs, and subscales if applicable."*

The development of the NEBS should be in the methods section and not the introduction.

                - *The development of the NEBS has now been moved to the methods section.*

The methods sections would benefit from traditional subheadings.

*-The methods sections now has subheadings.*

The results section contains demographic data which should be in the participant's section in the methods.

*-The participant demographic information was moved to the participant’s section in the methods.*

An exploratory factor analysis would have been useful on the first sample.

*-Please see last item for full response of this comment.*

**For study one,** the CFA should come first followed by test retests.

*-The test-restest results have been moved so that the CFA results come first followed by the test-retest results.*

Double-check the fit indices (particularly the CFI, the cut of is listed as .90 when in fact it should be .95) and was the chi-square significant?

*- These fit indices were taken from Cornell University Cornell Statistical Consulting Unit. Reference - Perry S: Fit Statistics commonly reported for CFA and SEM. Cornell University: Cornell Statistical Consulting Unit.2019.*

*-Yes, the chi-square was significant and has been added to the manuscript.*

**Study two –** why are there wellbeing measures? They are only mentioned briefly via a table in the results.

*-The well-being measures were included to test divergent validity. This is mentioned in the methods section. “* *Relevant measures used to establish NEBS divergent validity, which tests whether concepts or measurements that are not supposed to be related are actually unrelated were: Arizona Integrative Outcomes Scale* *^54^* *, Positive and Negative Affective Well-being Scale* *^55^* *, single-item general health* *^56^* *, acute sleep quality scale* *^57^* *, the Numeric Pain Rating Scale* *^58^* *, and Big Five Inventory-10 scale* *^59^* *, and the compassion subscale of the Dispositional Positive Emotion Scale.” The manuscript then goes on to describe each of those measures in more detail.*

The items relating to experience are all the same.

*-The wording of the experience items are the same but reflect the belief construct expressed in the item directly before.*

The participant is asked if they have experienced something directly after they have been asked if they believe it; this could present a confound.

*-Please see response below about EFA and dependencies between belief and experience items.*

Overall, while this paper offers an interesting premise, there are several flaws. The scale itself does not have dedicated experience items that refer to specific phenomena, which is problematic. The methodology itself is also flawed. I suspect an Exploratory Factor Analysis (EFA) would reveal that the factors would be around the phenomena rather than the dichotomy of belief and experience. I.e., a person, who believes in particular phenomena is more likely to experience them; therefore, the factors will demonstrate this. I would recommend that this be more of an exploratory study and that an EFA be run on the first sample at the very least. However, this would probably not give the desired outcome. The items that relate to experience should ideally be able to be answered in isolation and not be dependent on the belief items. This could act as a prime, with people who state they believe in something when asked if they have experienced it directly after being more likely to agree.

- *Exploratory factor analysis (EFA) is a statistical technique used to find the underlying structure of a set of observed variables (Gorsuch, 2015), whereas confirmatory factor analysis (CFA) is used when researchers have formulated a hypothesis regarding the relationship between observed variables and the underlying latent factors (Gorsuch, 2015).  There has been a debate regarding the circumstances in which these two analyses should be used in research (Hurley et al., 1997) and whether/when they should be used in tandem (Gerbing and Hamilton, 1996).  EFA can be used prior to cross-validation with CFA for the purpose of model specification (Gerbing and Hamilton, 1996).  EFA can also be used after CFA to explore poor fits in CFA models, explore factor structures when the original hypotheses are weak, and confirm factor structures when the original hypotheses were strong, but certain assumptions are not reasonable (Schmitt, 2011).*

*The reviewer recommends an EFA on the first sample.  It is suggested, but not made clear, that an EFA on the first sample would serve the purpose of exploration regarding other possible factor structures.  It is also suggested that EFA could possibly identify elevated correlation in the already correlated belief and experience factors that is due to survey structure alone.*

*In this study, hypotheses are based on theory and practice, and are therefore strong.  It is unclear whether EFA is suggested to confirm assumptions that may not hold in this study.  If so, what are these specific assumptions and why are they unreasonable or not upheld?  If EFA is suggested as a pre-cursor to CFA, then EFA should be conducted followed by cross-validation with CFA on an independent data set, as suggested by Gerbing and Hamilton (1996).  If this procedure is followed, either (1) the EFA will support the researchers’ hypotheses or (2) the EFA will not support the researchers’ hypotheses.  If (2) occurs, it is unclear what impact this should have on the current study.  The reviewer notes that if (2) occurs, “this would probably not give the desired outcome”, and it is highly pertinent to point out that the researchers did not undertake this study to achieve a “desired outcome” but rather to test hypotheses.  If (2) occurs, it is not clear whether this invalidates NEBS as a functioning survey tool.  CFA does not always confirm a factor structure obtained via EFA (van Prooijen and van der Kloot, 2001; Borkenau and Ostendorf, 1990).  If a CFA fits well and satisfies all assumptions but an EFA indicates that the underlying structure of the data can be represented by different factors, is this sufficient evidence to discard the original CFA?  If so, can the reviewer provide support for this claim?  In Gorsuch’s classic text on factor analysis, he states “Confirmatory factor analysis tests hypotheses that a specified subset of variables legitimately define a prespecified factor” (Gorsuch, 2015).  If the researchers have found that a subset of variables **legitimately** defined their prespecified factors and this was the central goal of their paper, what then is the purpose and role of added exploratory analysis in the context of this paper?*

*If the reviewer’s hypothesis that an EFA “would reveal that the factors would be around the phenomena rather than the dichotomy of belief and experience” is supported by an EFA on the first sample, then it is not clear whether presenting items in isolation would remedy this given the high inherent correlation between belief and experience, and therefore between the items themselves.  If this statement is more than opinion or a hypothesis and there is scientific support for it, how should this isolation be achieved and how much isolation is enough isolation to guarantee that this effect is removed from the analysis?  If the diagnosis of this specific issue is the only reason for the suggested EFA, then if the researchers were to reorganize and re-administer the surveys according to the reviewer’s specifications, would an EFA still be necessary?  It is also worth noting that the question of whether or not the survey structure introduced added dependence between items can be easily tested experimentally by providing the original and modified surveys (with some reasonable span of time in between) to a group of (new) participants and quantifying the difference in responses.  If the difference is not statistically significant, then any added dependence should be negligible.  Would this kind of adjustment in the methodology remedy the need for an EFA, according to the reviewer?*

*In summary, it is unclear whether the reviewer calls for an EFA to 1) examine other factor structures in the data or 2) test additional dependencies that may have been introduced by the survey structure.  If (1), it is unclear what role this EFA would play in the current paper.  If (2), it may be more straightforward to address this experimentally.*

**Literature Cited**

Borkenau, Peter and Ostendorf, Fritz (1990).  Comparing exploratory and confirmatory factor analysis: A study on the 5-factor model of personality. *Personality and Individual Differences*, 11(5), 515 – 524.

Gerbing, David W. and Hamilton, Janet G. (1996).  Viability of Exploratory Factor Analysis as a Precursor to Confirmatory Factor Analysis. *Structural Equation Modeling*, 3(1), 62 – 72.

Gorsuch, Richard L. (2015) Factor Analysis, Classic Edition.  Routledge: New York and London.

Hurley, Amy E., Scandura, Terri A., Schriesheim, Chester A., Brannick, Michael T., Seers, Anson, Vandenbery, Robert J., and Williams, Larry J. (1997).  Exploratory and confirmatory factor analysis: guidelines, issues, and alternatives. *Journal of Organizational Behavior*, 18, 667 – 683.

Schmitt, Thomas A. (2011).  Current Methodological Considerations in Exploratory and Confirmatory Factor Analysis. *Journal of Psychoeducational Assessment*, 29(4), 304 – 321.

Van Prooijen, Jan-Willem and van der Kloot, Willem A. (2001).  Confirmatory analysis of exploratively obtained factor structures. *Educational and Psychological Measurement*, 61(5):777 – 792.
